# Spontaneous Coronary Artery Dissection: An Unusual Cause of ST-Elevation Myocardial Infarction in Young Males

**DOI:** 10.7759/cureus.12827

**Published:** 2021-01-20

**Authors:** Tanvir Rahman, Reihaneh Moghadam, Morton Rinder

**Affiliations:** 1 Internal Medicine, St Luke's Hospital, Chesterfield, USA; 2 Internal Medicine, St. Luke's Hospital, Chesterfield, USA; 3 Cardiology, St. Luke's Hospital, Chesterfield, USA

**Keywords:** fmd, scad, pci, acs

## Abstract

Spontaneous coronary artery dissection (SCAD) is a non-traumatic, non-iatrogenic, and non-atherosclerotic coronary artery disorder that manifests clinically as an acute coronary syndrome (ACS), arrhythmia, or sudden cardiac death (SCD). It is a rare cause of ACS (1.7-4%) and SCD (0.5%), more commonly in women than men. It is rarely reported in males. We report a case of acute ST-elevation myocardial infarction (STEMI) due to SCAD in a 44-year-old healthy male.

## Introduction

Spontaneous coronary artery dissection (SCAD) is a non-traumatic, non-iatrogenic, and non-atherosclerotic coronary artery disorder that manifests clinically as an acute coronary syndrome (ACS), arrhythmia, or sudden cardiac death (SCD). It is a rare cause of ACS (1.7-4%) [1] and SCD (0.5%) [2]. SCAD is more commonly reported in young women <60 years of age, accounting for 22% to 35% of cases with a strong association with the peripartum period [3]. It was first reported in 1931 in a 42-year-old female at autopsy who had SCAD after violent retching and vomiting [4].

Once considered a rare occurrence, the prevalence of SCAD could be higher than previously thought. With the advent and widespread use of coronary angiography and optical coherence tomography (OCT), SCAD is being detected more than ever before. A recent study showed the occurrence of SCAD in 4% of cases out of 326 patients, who underwent routine OCT [1]. Another study by Vanzetto et al. showed the prevalence of SCAD at 8.7% in women less than 50 years of age, who presented with ACS [5]. It is important to note that the study analyzed only pregnancy-related MI and most of them occurred during the last-trimester or post-partum, affirming a strong correlation between SCAD and pregnancy. The mean age of the SCAD population was 44±9 years, and 92% were women with low rates of atherosclerotic risk factors. In the study by Tweet et al., five out of sixty-four (8%) patients had a major cardiac event [6,7]. Recent case series suggest women accounted for over 90% of cases of SCAD [8], reinstating the rarity of SCAD in males, and possibly underdiagnosed in males due to lower suspicion as it is most commonly related to pregnancy. In terms of racial distribution, the majority of the population suffered from SCAD were of Caucasian origin [9]. There are some recent case reports mentioning SCAD in the Asian population [10,11]. Here, we report a case of acute ST-elevation myocardial infarction (STEMI) due to SCAD in a 44-year-old healthy male.

## Case presentation

The patient experienced sudden-onset severe exertional, compressive, non-radiating chest pain. The pain did not resolve with rest or non-steroidal anti-inflammatory drugs (NSAIDs). The pain was associated with mild shortness of breath. The pain started the night prior, while he was cleaning his outdoor grill. Earlier that day, he moved heavy furniture around the household. He was an avid cyclist, practiced yoga, and martial arts regularly. Past medical history was negative for coronary artery disease (CAD), hypertension, hyperlipidemia, diabetes, or any connective tissue disorder. He complained of one episode of right lower extremity pain in 2016. A stress electrocardiogram (EKG) at that time was suggestive of ischemia, but it was subsequently ruled out with a negative stress echocardiogram. Family history was negative for any musculoskeletal, autoimmune, cardiac disorder, or sudden cardiac death. No history of smoking, or use of illicit drugs. He had no history of heavy alcohol consumption. The patient never used testosterone or any hormonal supplements. His only medication was Ibuprofen as needed for occasional knee joint pain from strenuous exercise. He is an immigrant from Southeast Asia. He was a computer engineer by profession, married, and has kids. No hyper-extensibility of skin and joints, no kyphosis, or scoliosis. 

**Figure 1:**
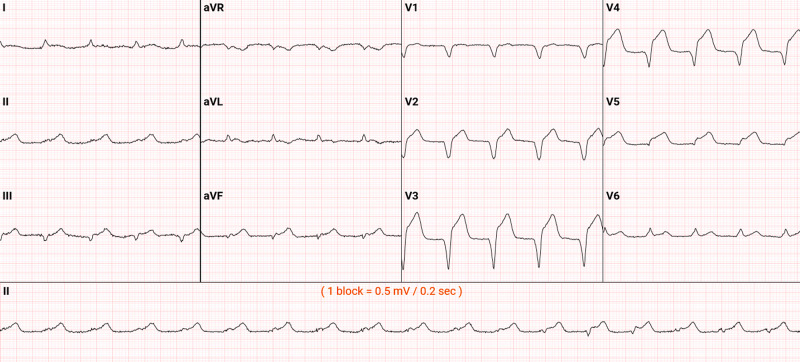
EKG on admission showing extensive ST-segment elevation in precordial leads (V2-V5) EKG: electrocardiogram.

In the field, EKG showed anterior-apical STEMI. Overt ST-segment elevation was noted in the chest leads, V2-V5 (Figure 1). He was treated with loading dose Aspirin (ASA) and sublingual nitroglycerin. The cardiac cath lab was activated immediately and the patient was brought to the emergency room (ER). Troponin was highly elevated at 52.4 ng/mL on hospitalization.

**Figure 2:**
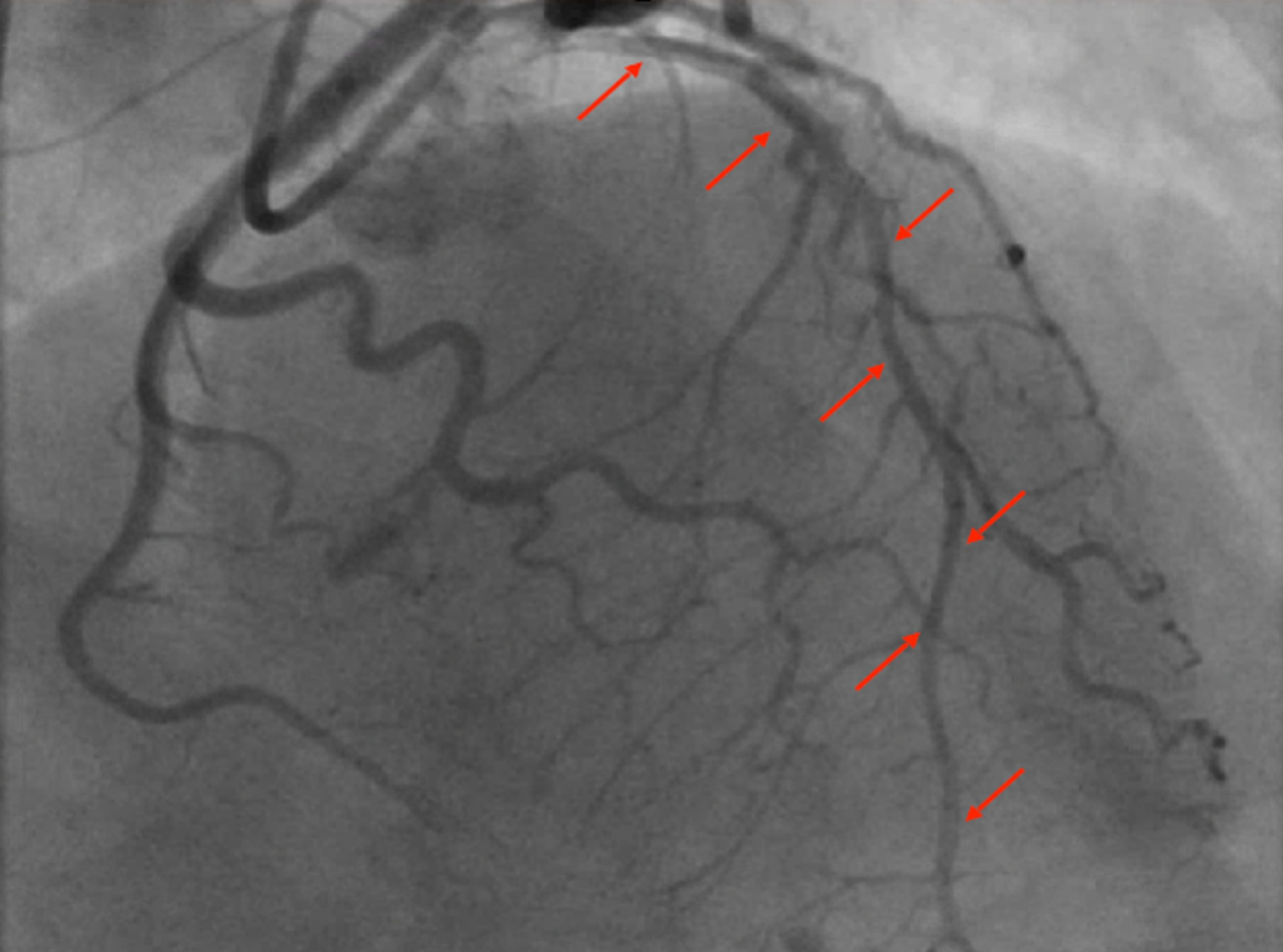
Extensive distal LAD dissection to 0% LAD: left anterior descending.

Emergent left heart catheterization (LHC) with coronary angiography revealed left dominant coronary artery circulation. The left main coronary artery revealed luminal irregularities throughout the vessel. The distal portion of the artery contained 95% stenosis and dissection of the mid into the distal vessel (Figure 2).

**Figure 3:**
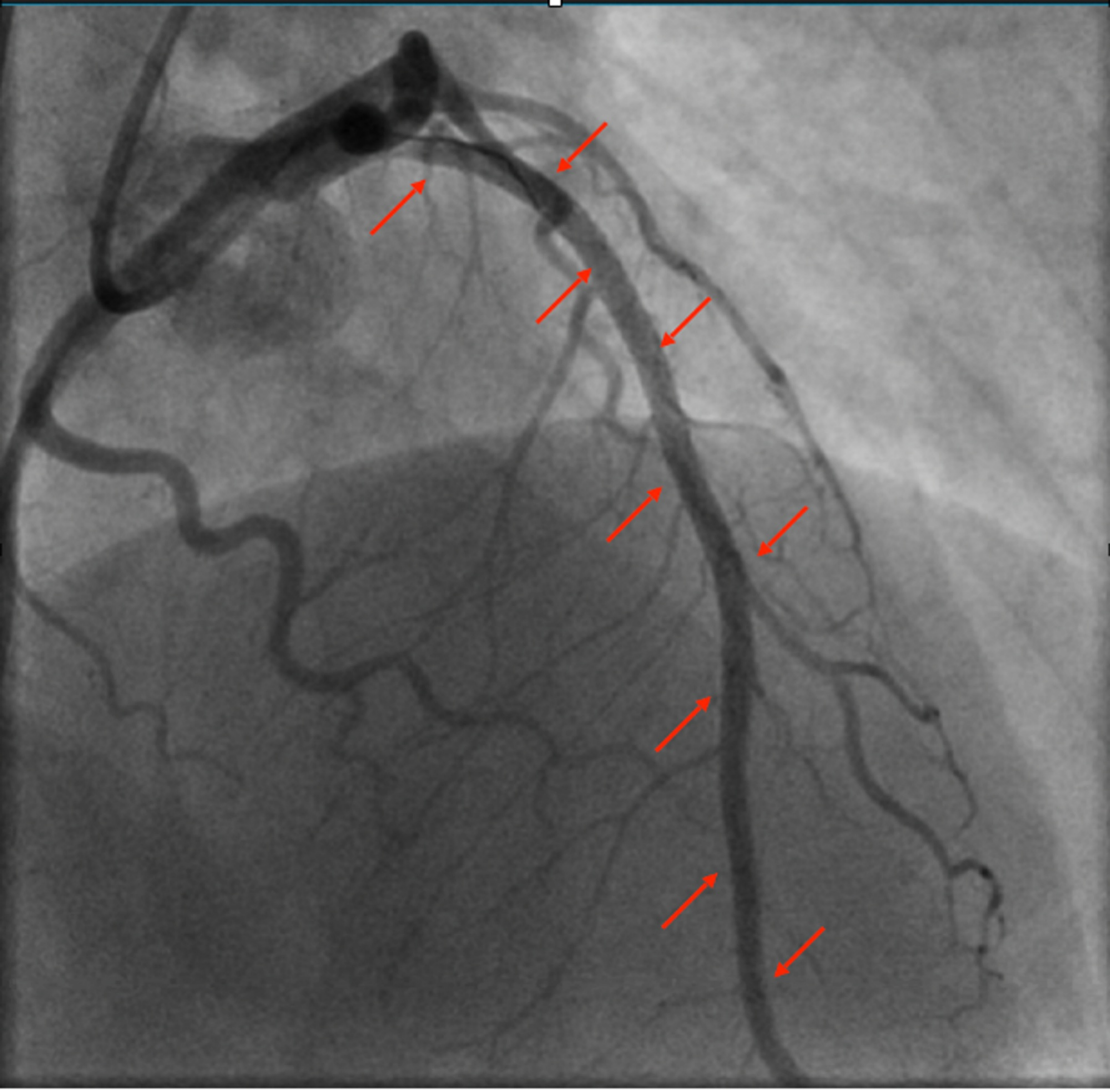
Successful PCI with DES × 4 with the restoration of TIMI flow DES: drug-eluting stents, PCI: percutaneous coronary intervention, TIMI: thrombolysis in myocardial infarction.

The diagonal artery/arteries revealed luminal irregularities throughout the vessel. Dissection may have had involved the second diagonal as well. The circumflex, obtuse marginal, ramus, and right coronary artery (RCA) revealed luminal irregularities throughout the vessel as well but no dissection was appreciated. Hyperdynamic base with severe hypokinesis of the mid to distal anterior wall and apex was noted. The successful percutaneous coronary intervention (PCI) of the distal left anterior descending (LAD) artery with a stenosis of 95% and thrombolysis in myocardial infarction (TIMI) flow of 2 was done. The lesion was of high complexity. Synergy 2.5 × 38 mm^2^ drug-eluting stent overlapped with a Synergy 2.75 × 32 mm^2^ drug-eluting stent overlapped with a Synergy 3.0 × 20 mm^2^ drug-eluting stent overlapped with a Synergy 2.75 × 16 mm^2^ drug-eluting stent (Figure 3). Post-PCI stenosis was 0% with a TIMI flow of 3. EF on transthoracic echocardiography (TTE) was 34% two-days after the LHC.

His chest pain resolved post-catheterization. Troponins trended down over the next few days. The patient was discharged home on ASA, statins, and metoprolol along with further follow-up instructions.

**Figure 4:**
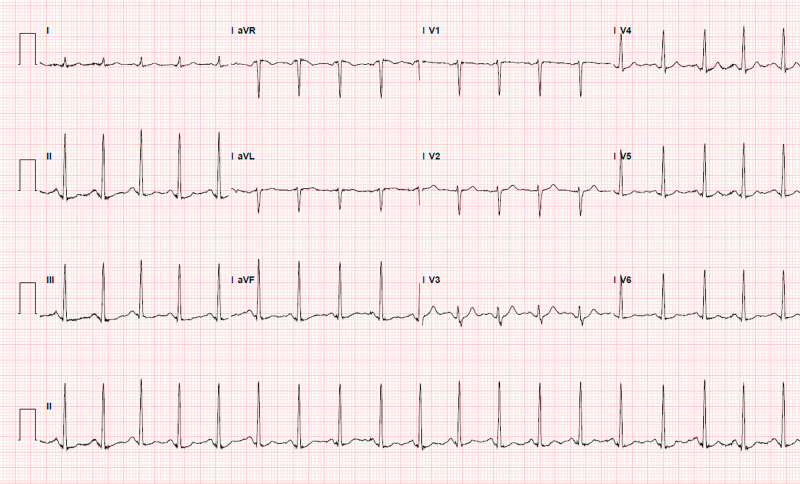
Lexiscan showing no EKG evidence of ischemia. The resting apical wall motion abnormality is consistent with the old anterior-apical MI. EKG: electrocardiogram.

Further physical examination was negative for collagen vascular disorders (e.g., Marfan syndrome, Ehlers Danlos syndrome). Autoimmune workup was negative. Magnetic resonance angiography (MRA) of head and neck without contrast was negative for fibromuscular dysplasia (FMD). Repeat TTE one-month post-SCAD showed an improvement of EF to 46% from 34%. Lexiscan performed six-months post-SCAD was non-revealing, apart from the signs of old infarction (Figure 4). He was doing well six-month post-MI with no recurrence of symptoms.

## Discussion

Due to the heightened suspicion of SCAD among physicians and the widespread use of cardiac catheterization, it is believed that the incidence of SCAD is higher than previously thought. The exact pathophysiology of SCAD is poorly understood. Two hypotheses predominate. The first hypothesis suggests spontaneous bleeding within the arterial wall, which creates a false lumen and ultimately obliterates the true lumen causing coronary insufficiency and MI. The other hypothesis points towards the rupture of vasa vasorum and ultimately culminating in ACS [12]. 

The most obvious predisposing factor in our patient was excessive physical exertion. No association with FMD, connective tissue, or autoimmune disorder was found, labeling the case as of idiopathic origin. FMD is frequently reported as an important risk factor for SCAD [13]. Association of SCAD and extra coronary FMD was first reported by Saw et al. in a case series in 2011 [13]. It is thought that FMD weakens the arterial wall and makes it more prone to aneurysms and dissections [14]. Histology-proven coronary FMD and SCAD were reported in some case reports, further supporting the association between SCAD and FMD [15,16]. Connective tissue disorders like Marfan and Ehlers Danlos syndrome (type 4) have been reported to be associated with SCAD but in 1-2% of cases [17].

In this case, the patient reported moving heavy furniture earlier that day from the basement to the upstairs by himself. He also reported cleaning his heavy outdoor grill on his porch in the middle of the cold weather. He also reported to bike 20 miles earlier that week. He is a trained martial artist and practiced martial arts and yoga routinely prior to his unfortunate presentation. Fahmy et al. reported isometric exercise as a predisposing factor to SCAD [18]. Physical stressors were high in men and emotional stressors were high in women as a predisposing factor to cause SCAD [18].

**Figure 5:**
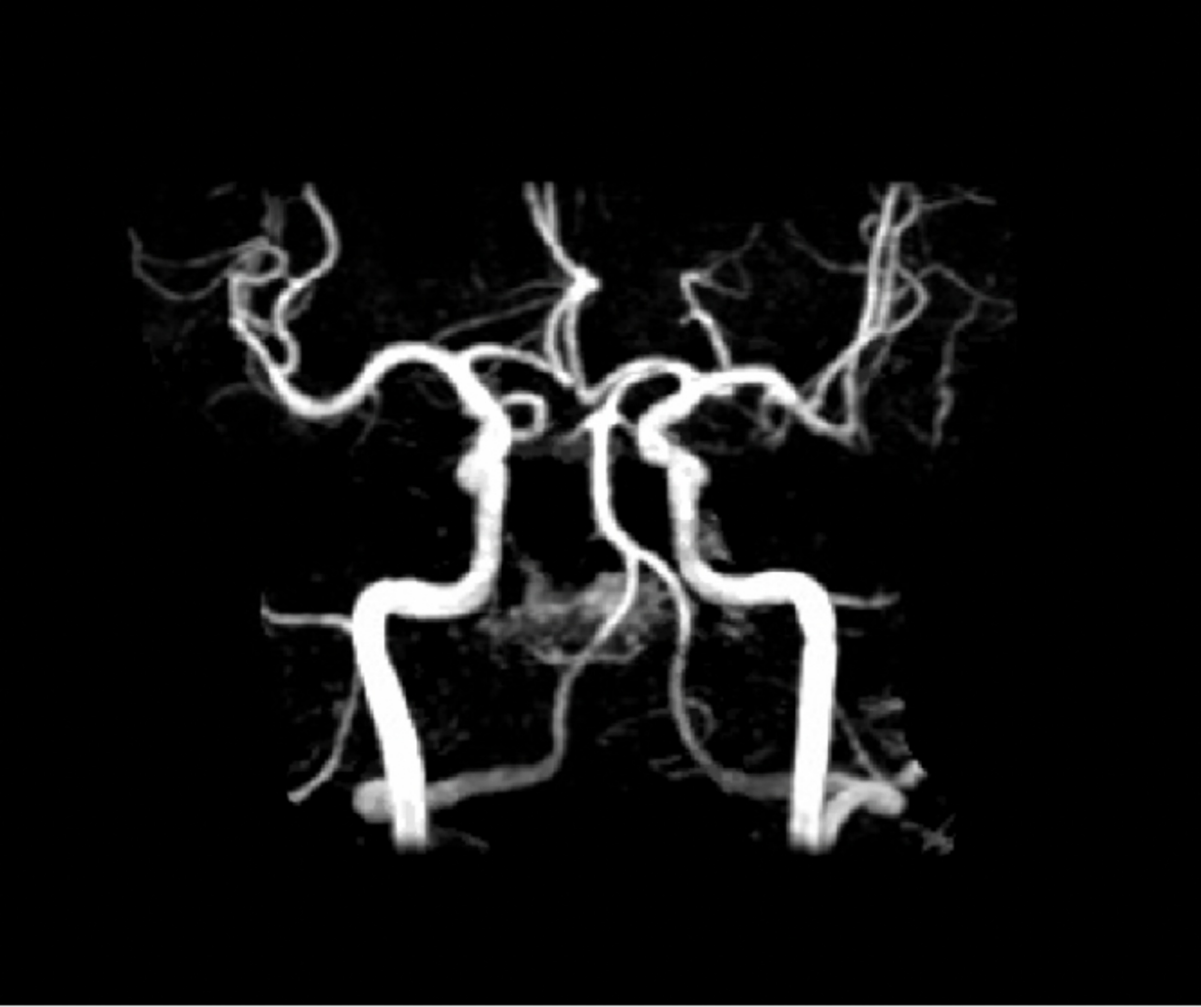
MRA head and neck without contrast was negative for typical bead-like pattern seen in FMD MRA: magnetic resonance angiography, FMD: fibromuscular dysplasia.

In the case of our patient, he presented with sudden-severe chest pain and with mild shortness of breath. To search for FMD, we ordered an MRA of head and neck without contrast, which showed normal-appearing cerebral arteries and was negative for FMD (Figure 5). CT angiogram (CTA) of renal arteries and pelvic vessels could not be done due to insurance issues and financial burden on part of the patient.

Any coronary artery could be affected in SCAD. Data from metanalysis showed involvement of LAD in 32-46%; left circumflex (LCX), ramus, and obtuse marginal in 15-45%; RCA and acute marginal, posterior descending (PD), posterolateral branches in 10-39%; and left main in 4% of cases [3,6,7].

There are three types of SCAD according to the Saw angiographic classification: type 1/pathognomic angiographic appearance with contrast staining of the arterial wall, type 2/diffuse stenosis of varying severity and length, and type 3/focal or tubular stenosis which mimics atherosclerosis [19].

Management of SCAD differs significantly from that of atherosclerotic ACS. Medical management with antiplatelet therapy, statins, beta-blockers, angiotensin-converting enzyme inhibitor (ACEI)/angiotensin II receptor blockers (ARBs), and anti-anginal therapy is suggested in SCAD patients who are hemodynamically stable and where major coronary arteries are not involved [20]. Conservative managements have shown better outcomes when compared to PCI in a medically stable patient, giving time to naturally heal the SCAD.

PCI with stents are recommended if the patient is unstable, or any major coronary is involved. CABG is reserved for left main dissection or severe proximal two-vessel dissection [20]. Our patient received a stent in the LAD and continues to be symptom-free to date. Transthoracic echocardiogram showed improvement of LV function which was 36% on admission and improved to 40% at six months follow-up.

The pathophysiology of SCAD is poorly understood adding up more challenges in diagnosis and management. Further epidemiological and large-scale prospective studies are essential to further elucidate the cause of SCAD to better aid in terms of diagnosis and treatment.

## Conclusions

This case is worthwhile reporting as SCAD is not usually reported as a cause of STEMI in adult males. A case like this demands more suspicion and awareness to recognize SCAD in a young male presenting with sudden severe chest pain with no significant CAD risk factors. Vigorous physical activity could be directly linked to this patient’s unfortunate diagnosis.
